# Increased Systemic Glucose Tolerance with Increased Muscle Glucose Uptake in Transgenic Mice Overexpressing RXRγ in Skeletal Muscle

**DOI:** 10.1371/journal.pone.0020467

**Published:** 2011-05-31

**Authors:** Satoshi Sugita, Yasutomi Kamei, Fumiko Akaike, Takayoshi Suganami, Sayaka Kanai, Maki Hattori, Yasuko Manabe, Nobuharu Fujii, Takako Takai-Igarashi, Miki Tadaishi, Jun-Ichiro Oka, Hiroyuki Aburatani, Tetsuya Yamada, Hideki Katagiri, Saori Kakehi, Yoshifumi Tamura, Hideo Kubo, Kenichi Nishida, Shinji Miura, Osamu Ezaki, Yoshihiro Ogawa

**Affiliations:** 1 Department of Molecular Medicine and Metabolism, Medical Research Institute, Tokyo Medical and Dental University, Tokyo, Japan; 2 Laboratory of Pharmacology, Faculty of Pharmaceutical Sciences, Tokyo University of Science, Chiba, Japan; 3 Graduate School of Human Health Sciences, Tokyo Metropolitan University, Tokyo, Japan; 4 Department of Bioinformatics, Graduate School of Biomedical Science, Tokyo Medical and Dental University, Tokyo, Japan; 5 Nutritional Science Program, National Institute of Health and Nutrition, Tokyo, Japan; 6 Department of Nutritional Science, Faculty of Applied Bioscience, Tokyo University of Agriculture, Tokyo, Japan; 7 Research Center for Advanced Science and Technology, University of Tokyo, Tokyo, Japan; 8 Department of Metabolic Diseases, Center for Metabolic Diseases, Tohoku University Graduate School of Medicine, Miyagi, Japan; 9 Department of Medicine, Metabolism and Endocrinology, School of Medicine, Juntendo University, Tokyo, Japan; 10 Sportology Center, Juntendo University, Tokyo, Japan; 11 Daiichi-Sankyo Co., Ltd., Tokyo, Japan; 12 Global Center of Excellence Program, International Research Center for Molecular Science in Tooth and Bone Diseases, Medical Research Institute, Tokyo Medical and Dental University, Tokyo, Japan; University of Tor Vergata, Italy

## Abstract

**Background:**

Retinoid X receptor (RXR) γ is a nuclear receptor-type transcription factor expressed mostly in skeletal muscle, and regulated by nutritional conditions. Previously, we established transgenic mice overexpressing RXRγ in skeletal muscle (RXRγ mice), which showed lower blood glucose than the control mice. Here we investigated their glucose metabolism.

**Methodology/Principal Findings:**

RXRγ mice were subjected to glucose and insulin tolerance tests, and glucose transporter expression levels, hyperinsulinemic-euglycemic clamp and glucose uptake were analyzed. Microarray and bioinformatics analyses were done. The glucose tolerance test revealed higher glucose disposal in RXRγ mice than in control mice, but insulin tolerance test revealed no difference in the insulin-induced hypoglycemic response. In the hyperinsulinemic-euglycemic clamp study, the basal glucose disposal rate was higher in RXRγ mice than in control mice, indicating an insulin-independent increase in glucose uptake. There was no difference in the rate of glucose infusion needed to maintain euglycemia (glucose infusion rate) between the RXRγ and control mice, which is consistent with the result of the insulin tolerance test. Skeletal muscle from RXRγ mice showed increased Glut1 expression, with increased glucose uptake, in an insulin-independent manner. Moreover, we performed *in vivo* luciferase reporter analysis using *Glut1* promoter (*Glut1*-Luc). Combination of RXRγ and PPARδ resulted in an increase in *Glut1*-Luc activity in skeletal muscle *in vivo*. Microarray data showed that RXRγ overexpression increased a diverse set of genes, including glucose metabolism genes, whose promoter contained putative PPAR-binding motifs.

**Conclusions/Significance:**

Systemic glucose metabolism was increased in transgenic mice overexpressing RXRγ. The enhanced glucose tolerance in RXRγ mice may be mediated at least in part by increased Glut1 in skeletal muscle. These results show the importance of skeletal muscle gene regulation in systemic glucose metabolism. Increasing RXRγ expression may be a novel therapeutic strategy against type 2 diabetes.

## Introduction

The skeletal muscle, known as the largest organ in the human body, plays an important role in exercise, energy expenditure, and glucose metabolism. It is a major site of glucose disposal [1], [2]. In type 2 diabetic subjects, glucose uptake in the skeletal muscle is impaired [2]. Blood glucose is taken up by the skeletal muscle via insulin-dependent and independent glucose transporters (Glut4 and Glut1, respectively) [3], where it is converted into glycogen. Increasing capacity of glucose uptake in the skeletal muscle is considered beneficial for the prevention and treatment of type 2 diabetes [3], [4]. On the other hand, glucose metabolism in the skeletal muscle may affect the whole body metabolism; the skeletal muscle-specific inactivation of *Glut4* has resulted in defect in insulin action in the adipose tissue (glucose uptake) and liver (suppression of gluconeogenesis) [5]. Thus, identification of the molecular mechanisms involved in skeletal muscle glucose metabolism should help clarify the pathophysiology of diabetes.

Nuclear receptors are part of a large superfamily of transcription factors that includes receptors for steroids, retinoic acid, and thyroid hormones [6]. RXRs are heterodimeric partners of many nuclear receptors, such as retinoic acid receptors (RARs), thyroid hormone receptors (T3Rs), liver X receptors (LXRs), peroxisome proliferator activated receptors (PPARs), and RXRs themselves [6]. Among RXR heterodimer partners, activation of PPARδ in the skeletal muscle increases insulin sensitivity [7]. The RXR subfamily consists of RXRα, RXRβ, and RXRγ [6], [8]. Although RXRγ is preferentially expressed in the skeletal muscle, its functional role is poorly understood. We have recently found that expression of retinoid X receptor γ (RXRγ) is changed in the skeletal muscle under nutritional conditions; RXRγ mRNA expression is down-regulated by fasting and recovered by refeeding [9]. In an attempt to explore the role of RXRγ in the skeletal muscle, we established transgenic mice overexpressing RXRγ in the skeletal muscle (RXRγ mice) and found that they exhibit increased triglyceride contents in the skeletal muscle as a result of increased expression of sterol regulatory element binding protein 1c (SREBP1c), a transcriptional master regulator of lipogenesis [9]. Indeed, RXRγ has been shown to enhance SREBP1c gene expression in C2C12 myocytes *in vitro* at least in part by heterodimerization with LXR [9]. On the other hand, we also found that blood glucose levels are lower in RXRγ mice than in control mice [9]. These observations, taken together, suggest that RXRγ plays a critical role in glucose and lipid metabolism in the skeletal muscle. However, the molecular mechanism involved in RXRγ regulation of glucose metabolism in the skeletal muscle and how it affects systemic glucose metabolism are poorly understood.

Here, we demonstrate enhanced glucose metabolism with increased Glut1 expression and glucose uptake in RXRγ mice. This study suggests that activation of the skeletal muscle RXRγ is a novel therapeutic strategy to treat or prevent type 2 diabetes.

## Methods

### Animals

C57BL6 mice were purchased from Charles River Japan (Yokohama, Japan). Generation of RXRγ mice under the control of the human α-actin promoter was described previously [9]. They were allowed free access to food (CRF-1; Charles River) and water, unless otherwise stated. All animal experiments were approved by Institutional Animal Care and Use Committee of Tokyo Medical and Dental University (approval ID: No. 0090041).

### Blood analysis

Serum samples were obtained from mice, when fed *ad libitum*. Serum glucose levels were measured by the blood glucose test meter (Glutest PRO R; Sanwa-Kagaku, Nagoya, Japan). Serum concentrations of insulin were determined by the enzyme-linked immunosorbent assay (ELISA) kits (Morinaga Institute of Biological Science, Inc., Yokohama, Japan).

### Glucose and insulin tolerance tests

For glucose tolerance test, D-glucose (1 mg/g of body weight, 10% (w/v) glucose solution) was administered by intraperitoneal injection after an overnight fast. For insulin tolerance test, human insulin (Humulin R; Eli Lilly Japan K.K., Kobe, Japan) was injected intraperitoneally (0.75 mU/g of body weight), when fed *ad libitum*.

### Hyperinsulinemic-euglycemic clamp studies

We analyzed as described previously [10]–[12] with slight modification. Two days before the clamp studies, a catheter was inserted into the right jugular vein for infusion under general anesthesia with sodium pentobarbital. Studies were performed on mice under conscious and unstressed conditions after a 4-h fast. The clamp study began with a prime (4 mg/kg for 5 min) of [6,6-^2^H] glucose (Cambridge Isotope Laboratories, Inc, Andover, MA) followed by continuous infusion at a rate of 0.5 mg/kg per minute for 2 hr to assess the basal glucose turnover. After the basal period, hyperinsulinemic-euglycemic clamp was conducted for 120 min with a primed/continuous infusion of human insulin (25 mU/kg prime for 5 min, 5 mU/kg/min infusion) and variable infusion of 20% glucose to maintain euglycemia (approximately 100 mg/dl). The 20% glucose was enriched with [6,6-^2^H] glucose to approximately 2.5% as previously described [12]. To determine the enrichment of [6,6-^2^H]glucose in plasma at basal and insulin stimulated state, samples were deproteinized with trichloroacetic acid and derivatized with *p*-aminobenzoic acid ethyl ester. The atom percentage enrichment of glucose_m+2_ was then measured by high-performance liquid chromatography with LTQ-XL-Orbitrap mass spectrometer (Thermo Scientific, CA). The glucose_m+2_ enrichment was determined from the *m/z* ratio 332.2: 330.2. The hepatic glucose production was calculated by using the rate of infusion of [6,6-^2^H]glucose over the atom percent excess in the plasma minus the rate of glucose being infused. The insulin-stimulated whole-body glucose uptake was calculated by adding the total glucose infusion rate plus the hepatic glucose production [12].

#### Quantitative real-time PCR

Quantitative real-time PCR was performed as described [9]. Total RNA was prepared using Sepazol (Nacalai Tesque, Kyoto, Japan). cDNA was synthesized from 5 µg of total RNA using Superscript II reverse transcriptase (Invitrogen Inc., Carlsbad, CA) with random primers. Gene expression levels were measured with an ABI PRISM 7700 using SYBR Green PCR Core Reagents (Applied Biosystems, Tokyo, Japan). The primers used were as follows, RXRγ: Fw: 5′-CACCCTGGAGGCCTATACCA-3′, Rv: 5′-AAACCTGCCTGGCTGTTCC-3′, Glut1: Fw: 5′-CCAGC TGGGA ATCGT CGTT-3′, Rv: 5′-CAAGT CTGCA TTGCC CATGAT-3′, Glut4: Fw: 5′-TCTGTGGGTGGCATGATCTCT-3′, Rv: 5′-GCCCTTTTCCTTCCCAACC-3′, glucose phosphate isomerase 1: Fw: 5′-AGCGCTTCAACAACTTCAGCT-3′, Rv: 5′-CAGAATATGCCCATGGTTGGT-3′, phosphoglycerate mutase 1: Fw: 5′-TCCTGAAACATCTGGAAGGTATCTC-3′, Rv: 5′-CAGTGGGCAGAGTGATGTTGAT-3′, fructose bisphosphatase 2: Fw: 5′-TGAATGCAATCCTGTGGCC-3′, Rv: 5′-TGGTTGCCATACCTCCTGCT-3′, pyruvate dehydrogenase kinase isoenzyme 1: Fw: 5′-GGACTTCTATGCGCGCTTCT-3′, Rv: 5′-CTGACCCGAAGTCCAGGAAC-3′, glycogen synthase 2: Fw: 5′-AGGATCATTCAGAGGAACCGC-3′, Rv: 5′-CCAGTCCAGGAGATCTGAGAGC-3′.

### Tissue sampling for analysis of Glut1 and Glut4 protein levels

Skeletal muscles were homogenized in ice-cold buffer containing 250 mmol/L sucrose, 20 mmol/L 2-[4-(2-hydroxyethyl)-1-piperadinyl] ethonsulforic acid (HEPES) (pH 7.4), and 1 mmol/L EDTA, and centrifuged at 1200 g for 5 minutes. The supernatant was centrifuged at 200 000 g for 60 minutes at 4°C [13]. The resulting pellet was solubilized in Laemmli sample buffer containing dithiothreitol. Samples were subjected to Western blotting as described [14]. Antibodies used were those against Glut1 (#07-1401, Millipore, Temecula, CA), and Glut4 (SC-1606, Santa Cruz Biotechnology Inc., Santa Cruz, CA).

### Muscle incubation and glucose transport

Glucose transport was measured as described [15]. Mice were fasted overnight and killed. The extensor digitorum longus (EDL) muscles were rapidly removed, each of which was mounted on the incubation apparatus and preincubated in Krebs-Ringer bicarbonate (KRB) buffer containing 2 mmol/l pyruvate for 30 min. The muscles were then incubated in KRB buffer in the absence or presence of 50 mU/ml insulin for 10 min. The buffers were kept at 37°C throughout the experiment and gassed continuously with 95% O_2_ and 5% CO_2_. Immediately after incubation, the muscle was transferred to KRB buffer containing 1 mmol/l 2-[^3^H]-deoxy-d-glucose (1.5 µCi/ml) and 7 mmol/l d-[^14^C]-mannitol (0.3 µCi/ml).

### Measurement of skeletal muscle glycogen

The skeletal muscle glycogen content was measured as glycosyl units after acid hydrolysis [16]. The skeletal muscle samples were minced, 50 mg of which was added to 1 ml of 0.3 M percholic acid and homogenized on ice. One ml of 1 N HCl was added and incubated for 2 h at 100°C, thereafter, 1 ml of 1 N NaOH was added at room temperature. Glucose content was examined using the F-kit glucose (Roche Diagnostics, Mannheim, Germany).

### Plasmids

The coding region of mouse RXRγ and PPARδ cDNA were subcloned into a mammalian expression plasmid, pCMX [9]. The 1.5-kb 5′-flanking region of the mouse *Glut1* promoter was obtained by PCR with mouse genomic DNA (RefSeqs number: NC_000070). The PCR primers used were: Fw: 5′-GTGGTGCGCGCCTGTAGTCC-3′ and Rv: 5′-GGCGCACTCCACGGATGCCG-3′. The fragment was subcloned into the pGL3-basic luciferase vector (Promega Corporation, Madison, WI). The promoter regions used were: -1500 to +75, -647 to +75, and -152 to +75, counting the transcription start site as +1.

### Electroporation and *in vivo* luciferase reporter analysis

*In vivo* electroporation was performed according to the modified method of Aihara and Miyazaki [17]. Under the pentobarbital anesthesia (30 mg/kg), bilateral quadriceps muscles from C57BL6 mice (male, 12 weeks of age) were injected with 80 µg of plasmid DNA (25 µl) by using a 29-gauge needle attached to a 0.5-ml insulin syringe (Terumo Corporation, Tokyo, Japan). Square-wave electrical pulses (160 V/cm) were applied six times with an electrical pulse generator (CUY21EDIT, Nepa Gene Co. Ltd., Chiba, Japan) at a rate of one pulse per second, with each pulse being 20 ms. in duration. The electrodes were a pair of stainless steel needles inserted into the quadriceps muscles and fixed 5 mm apart. Seven days after gene delivery, the muscles were removed and subjected to analysis. Frozen muscle tissues were homogenized in ice-cold passive lysis buffer from Promega. The homogenate was centrifuged at 10,000 g for 10 min at 4°C. The supernatant was reserved for luciferase assay using Promega's dual luciferase assay kit. The luciferase activity was calculated as the ratio of firefly to Renilla (internal control) luciferase activity and represented as the average of triplicate experiments.

### Computer-based DNA sequence motif search

We used MATCH software [18] (BIOBASE GmbH, Wolfenbuettel, Germany) to investigate transcriptional binding sites in the mouse *Glut1* promoter. We investigated mouse genome in the region of -1500 to +100 relative to transcription start of *Glut1* (Chr.4 11878131(+)).

### cDNA microarray analysis

RNA was isolated from the skeletal muscle of sex- and age-matched RXRγ mice (line 4-3) and non-transgenic control mice (females at 4 months of age, five samples from each group were combined). Each of the combined samples was hybridized to the Affymetrix MG430 microarray, which contains 45,102 genes, including expressed sequence tags (ESTs), and analyzed with the software Affymetrix Gene Chip 3.1. Of the 45,102 genes including ESTs analyzed, 8,054 (non-transgenic control mice) and 8,083 (transgenic) were expressed at a substantial level (absolute call is present and average difference is above 150). In order of fold changes in gene expression levels in skeletal muscle from RXRγ mice relative to control mice, genes whose expression was increased more than 2 ^0.4^-fold in RXRγ mice were listed (Dataset S1). Fold changes were calculated as an indication of the relative change of each transcript represented on the probe array. Differentially expressed genes were identified using the following criteria; ‘absolute call’ is present, and ‘average difference’ was above 250. ‘Absolute call’, which was calculated with this software using several markers, is an indicator of the presence or absence of each gene transcript. The ‘average difference’ value is a marker of the abundance of each gene, obtained by comparing the intensity of hybridization to 20 sets of perfectly matched 25-mer oligonucleotides relative to 20 sets of mismatched oligonucleotides using Affymetrix Gene Chip 3.1 software. All data of microarray is MIAME compliant and that the raw data has been deposited in a MIAME compliant database (GEO), whose accession number is GSE28448.

### Gene Ontology Analysis

We used DAVID v6.7 [19] for gene ontology (GO) analysis. DAVID is a web application providing a comprehensive set of functional annotation tools to understand the biological meaning behind a large list of genes. Functional Annotation Clustering of DAVID was applied to the genes whose gene expression increased in RXRγ mice. Our GO analysis produced 62 GO terms from gene sets from RXRγ mice with increased expression compared to the wild-type mice, under the condition of *P*<0.05 (*P* value from Fisher's Exact Test). The obtained GO terms contained many similar functional concepts. In order to group similar GO terms, we applied the Functional Annotation Clustering tool provided by DAVID [19], [20]. Twenty-three clusters were produced with genes showing increased expression. We showed one GO term of the lowest *P* value of all the members of an individual cluster.

### Transcriptional factor binding sites analysis

We used MATCH software [18] with BKL TRANSFAC 2010.3 Release (BIOBASE GmbH, Worfenbuettel, Germany) to investigate transcriptional binding sites in the promoter regions of genes. The F-Match algorithm compares the number of sites found in a query sequence set against the background set. It is assumed, if a certain transcription factor (or factor family), alone or as a part of a *cis*-regulatory module, plays a significant role in the regulation of the considered set of promoters, then the frequency of the corresponding sites found in these sequences should be significantly higher than expected by random chance. We investigated the mouse genome in the region of -1000 to +100 relative to the transcription start of an individual gene. Statistical hypothesis testing was evaluated against housekeeping genes of mice. We investigated promoter regions of 15 genes: that is 14 ‘glucose metabolic process’ genes with increased expression in RXRγ mice, plus Glut1. In the GO analysis, Glut1 was categorized as a ‘transporter’ not ‘glucose metabolic process’ gene.

### Statistical analysis

Statistical analysis was performed using the Student's *t* test and analysis of variance (ANOVA) followed by Scheffe's test. Data were expressed as the mean ± SE. *P*<0.05 was considered statistically significant.

## Results

### Increased glucose metabolism in RXRγ mice

In our previous study, we established two lines of RXRγ mice (named lines 4-3 and 5-3) with similar expression levels of the *RXRγ* transgene and protein specifically in the skeletal muscle [9]. There was no significant difference in body weight, adipose tissue, and liver weight between RXRγ and control mice in both lines 4-3 and 5-3, although the skeletal muscle weight was slightly lower in RXRγ mice than in control mice (Tables 1 and 2). In this study, blood glucose in RXRγ mice was lower than in control mice (fasting state, *P*<0.01 in lines 4-3 and 5-3; basal state, *P*<0.05 in line 4-3 and *P* = 0.06 in line 5-3), which is consistent with our previous report [9].

**Table 1:** Body and dissected tissue weight and blood glucose level in RXRγ mice (4-3 line).

	Wild-type	RXRγ
Body weight (g)	26.4±0.3	26.5±0.4
Epididymal fat mass (g)	0.138±0.004	0.156±0.090
Gastrocnemius muscle weight (g)	0.282±0.004	0.238±0.008**
Liver weight (g)	1.290±0.029	1.321±0.033
Glucose (mg/dL) basal	170.0±9.4	145.6±4.9*
Glucose (mg/dL) fasting	74.4±1.7	67.0±0.6**

Mice were males 12 weeks of age. The number of animals used was 6 for both wild-type control and RXRγ mice.

* *P*<0.05;

** *P*<0.01, compared with wild-type control.

Values are the means ± SE. These samples were also used in Fig 2*A, B*, and *D*.

**Table 2:** Body and dissected tissue weight and blood glucose level in RXRγ mice (5-3 line).

	Wild-type	RXRγ
Body weight (g)	36.0±1.8	31.9±1.2
Epididymal fat mass (g)	1.030±0.191	0.861±0.134
Gastrocnemius muscle weight (g)	0.305±0.016	0.214±0.020**
Liver weight (g)	1.375±0.119	1.320±0.066
Glucose (mg/dL) basal	142.5±8.6	117.3±8.2
Glucose (mg/dL) fasting	67.2±1.6	53.3±2.5**

Mice were males 38 weeks of age. The number of animals used was 6 for both wild-type control and RXRγ mice.

** *P*<0.01, compared with wild-type control.

Values are the means ± SE.

**Figure 1:**
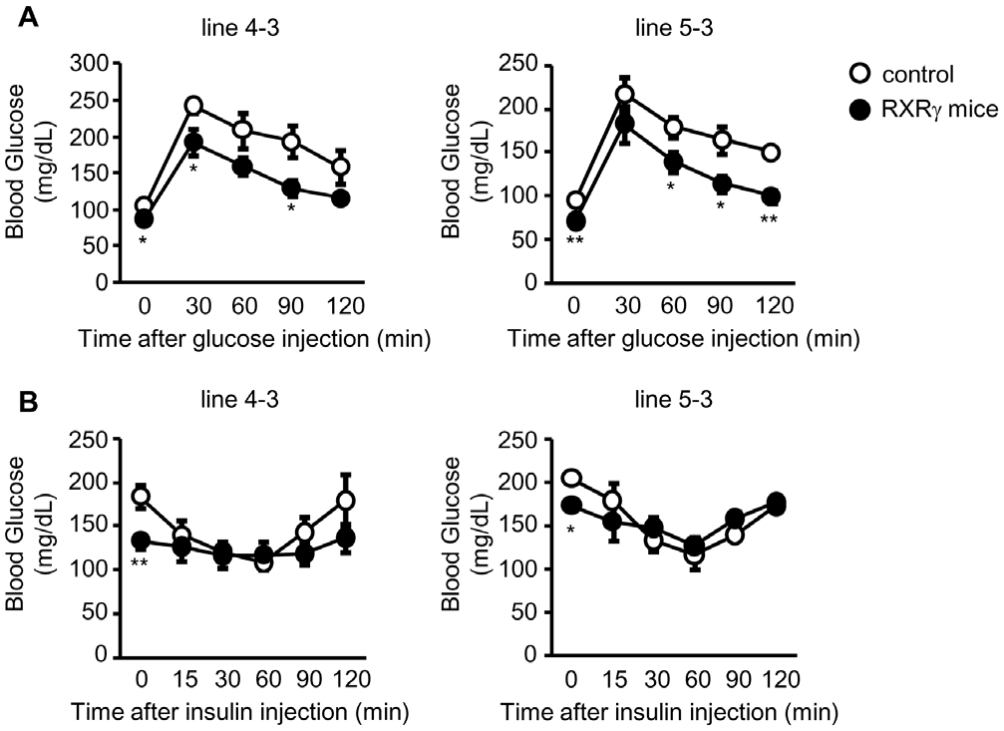
Glucose tolerance and insulin tolerance tests on RXRγ mice. (*A*, *B*) In A and B, male mice, 5 months of age, were used. The number of animals used was 6 for both control (open circles) and RXRγ (filled circles) mice of line 4-3, and 5 for both control (open circles) and RXRγ (filled circles) mice of line 5-3. * *P*<0.05 and ** *P*<0.01 compared with respective control.

To elucidate the role of the skeletal muscle RXRγ in systemic glucose metabolism, we performed glucose and insulin tolerance tests in RXRγ mice. The glucose tolerance test revealed increased glucose disposal in RXRγ mice relative to control mice (Fig. 1*A*). On the other hand, there was no significant difference in the insulin-induced hypoglycemic response between genotypes (Fig. 1*B*). These observations suggest that RXRγ mice have a higher capacity for glucose disposal with no change in insulin sensitivity.

### Increased Glut1 expression and glucose uptake in the skeletal muscle of RXRγ mice

**Figure 2:**
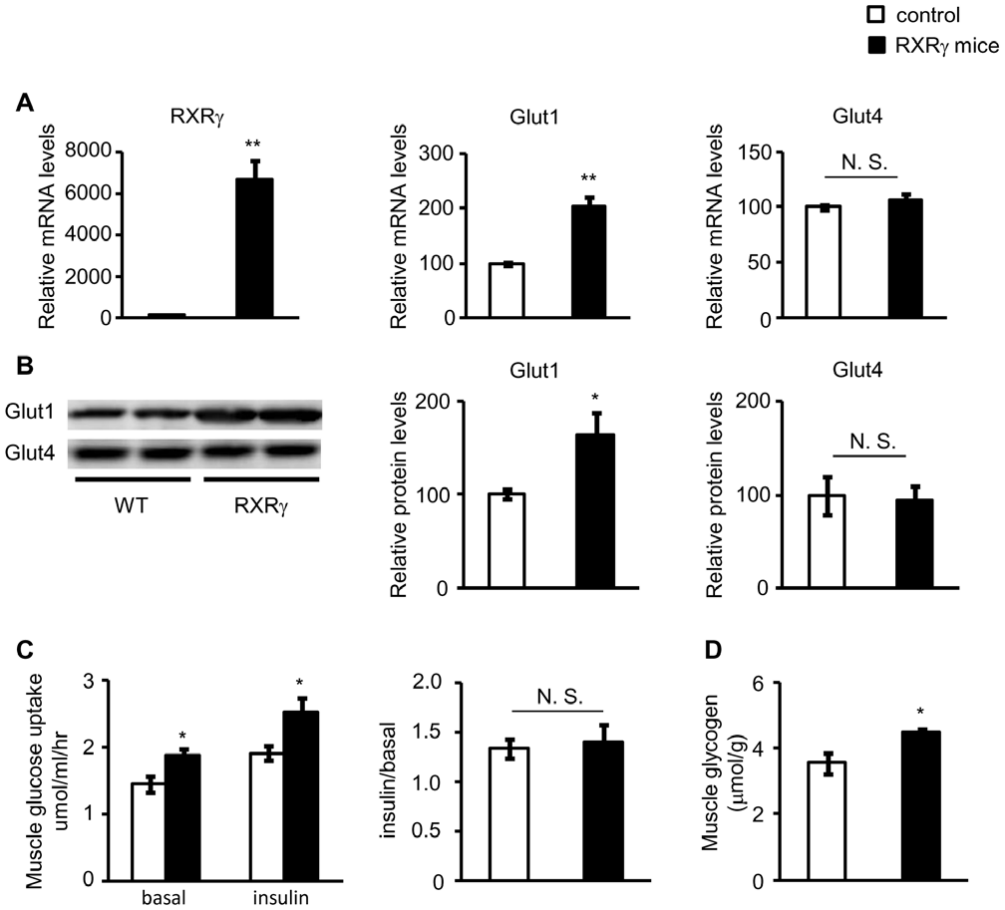
Levels of Glut 1 and Glut4, and glucose uptake in the skeletal muscle of RXRγ mice. (*A*) Gene expressions of *RXRγ*, *Glut1*, and *4* were examined by quantitative real-time PCR. The value for wild-type (littermates of line 4-3) mice was set at 100, and relative values are shown. (*B*) Protein levels of Glut1 and Glut4 were examined by Western blotting. Results of relative densitometric signal for Glut1 and 4 are shown. (*C*) Glucose uptake in the absence or presence of insulin and (*D*) glycogen content were increased in the skeletal muscle of RXRγ mice. Ratio of enhanced glucose uptake in the presence of insulin (insulin/basal) was similar in control and RXRγ mice. In *A*, *B* and *D*, the same samples were used. Mice were males of 12 weeks of age. The number of animals was 6 for both control (open bars) and RXRγ (filled bars) mice. These samples were also used in Table 1. In *C*, mice were males of 24–27 weeks of age. The number of animals was 6 for both control (open bars) and RXRγ (filled bars) mice. * *P*<0.05 and ** *P*<0.01 compared with respective control. N. S., not significant.

As both lines of RXRγ mice showed increased glucose metabolism, for the following experiments, we only utilized the line 4-3 of RXRγ mice (hereafter just RXRγ mice). Interestingly, mRNA expression of *Glut1* was increased in the skeletal muscle from RXRγ mice relative to control mice (*P*<0.01), whereas that of *Glut4* was unchanged (Fig. 2*A*). We also observed that Glut1 is significantly increased in RXRγ mice at the protein level (*P*<0.05), with no significant difference in Glut4 between genotypes (Fig. 2*B*). Consistently, we also found increased glucose uptake in the skeletal muscle from RXRγ mice relative to control mice (*P*<0.05), which was not further enhanced in the presence of insulin (Fig. 2*C*). We also observed increased glucose glycogen content in the skeletal muscle from RXRγ mice relative to control mice (*P*<0.05, Fig. 2*D*).

### Increased basal glucose disposal rate of RXRγ mice

**Figure 3:**
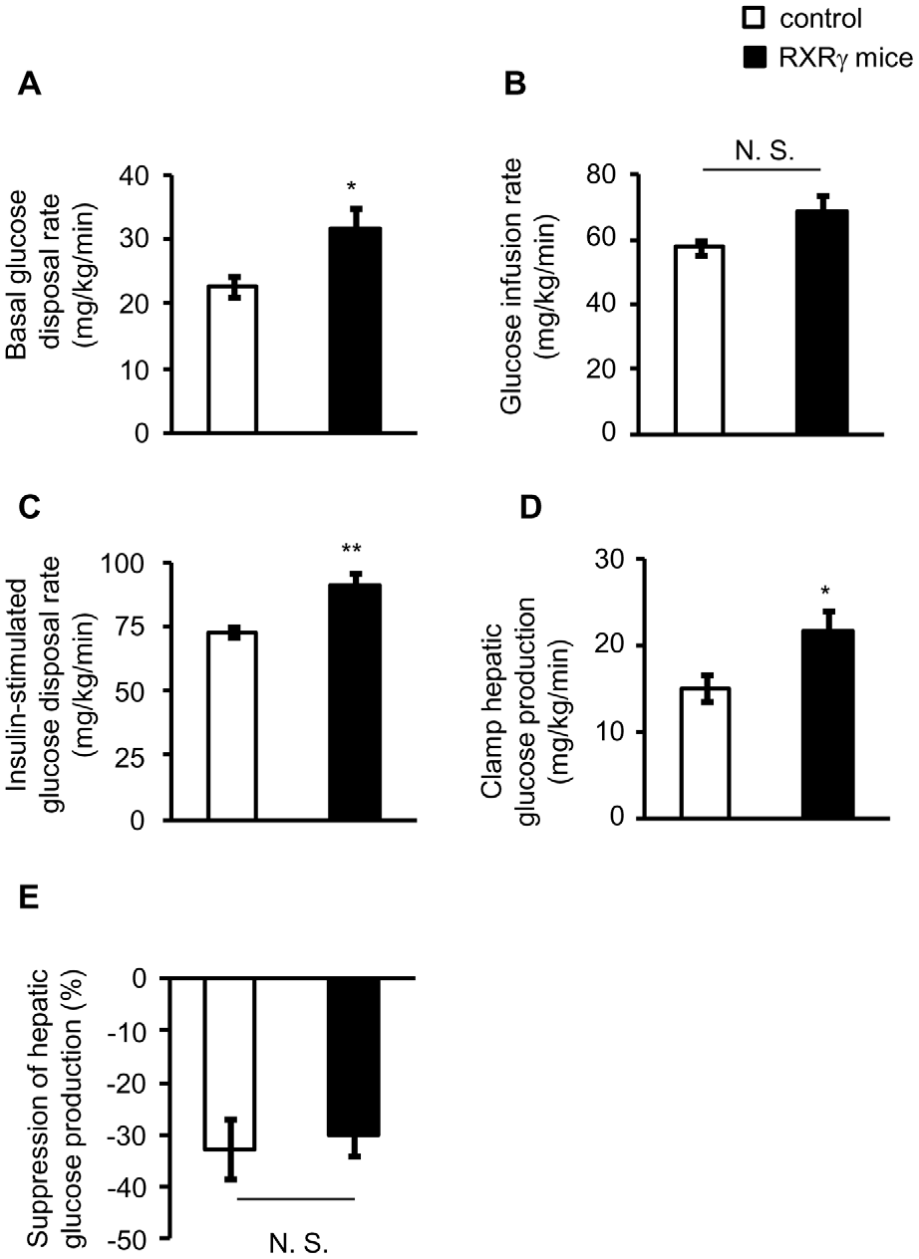
Hyperinsulinemic-euglycemic clamp test in RXRγ mice, fed a chow diet. (*A*) Basal glucose disposal rate, (*B*) glucose infusion rate needed to maintain euglycemia, (*C*) insulin-stimulated glucose disposal rate and (*D*) clamp hepatic glucose production (hepatic glucose production during the clamp period) (*E*) suppression of hepatic glucose production during the clamp period in RXRγ mice. Male mice, 13∼16 weeks of age, were used. The number of animals used was 6 for control mice (open bars) and 7 for RXRγ mice (filled bars). * *P*<0.05 and ** *P*<0.01 compared with respective control. N. S., not significant.

To gain further insight into the glucose metabolism in RXRγ mice, we performed a hyperinsulinemic-euglycemic clamp study. Plasma insulin concentrations during the basal period were similar between genotypes (Table 3). The basal glucose disposal rate was significantly increased in RXRγ mice than in the controls (*P*<0.05, Fig. 3*A*), supporting that an insulin-independent increase in glucose uptake occurred, as observed in Fig. 2*C*. Meanwhile, we observed that the rate of glucose infusion needed to maintain euglycemia (glucose infusion rate) was similar between genotypes (Fig. 3*B*), which is consistent with the result of the insulin tolerance test (Fig. 1*B*). On the other hand, insulin-stimulated glucose disposal rate was higher in RXRγ mice than in the controls (*P*<0.01, Fig. 3*C*), which probably reflects the increased basal glucose disposal rate (Fig. 3*A*). Also, the clamp hepatic glucose production (hepatic glucose production during the clamp period) was higher in RXRγ mice than in the controls (*P*<0.05, Fig. 3*D*). Meanwhile, hepatic glucose production was similarly suppressed by insulin in both genotypes (Fig. 3*E*). Together, these data support the idea that Glut1, an insulin-independent glucose transporter, is involved in the increased glucose disposal in the skeletal muscle of RXRγ mice.

**Table 3:** Body weight, blood glucose and plasma insulin levels in RXRγ mice in clamp study.

		Wild-type	RXRγ
	Body weight (g)	24.1±1.2	23.4±0.3
Basal period	Glucose (mg/dL)	141.8±15.3	106.6±5.5**
	Insulin (ng/mL)	0.55±0.08	0.44±0.07
Clamp period	Glucose (mg/dL)	92.5±3.8	96.7±3.6
	Insulin (ng/mL)	2.60±0.20	2.38±0.33

Mice were males 13–16 weeks of age. The number of animals used was 6 for wild-type control mice and 7 for RXRγ mice.

* *P*<0.05, compared with wild-type control.

Values are the means ± SE. These mice were also used in Fig 3.

### Activation of the *Glut1* promoter by combination of RXRγ and PPARδ in the skeletal muscle *in vivo*

**Figure 4:**
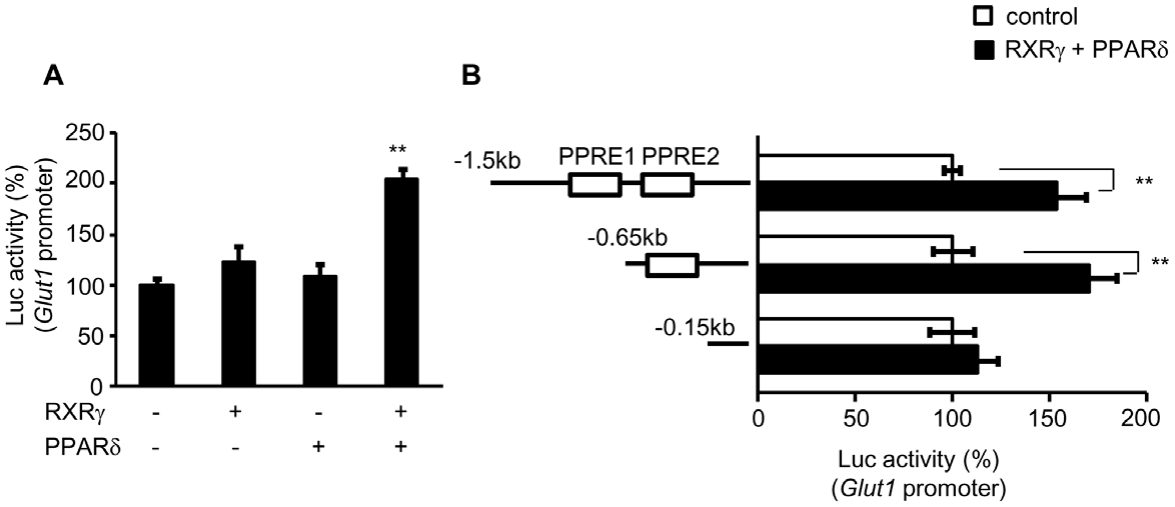
Transient transfection-reporter assay of the effect of RXRγ on *Glut1* promoter. (*A*) *Glut1*-Luc plasmid, with or without RXRγ and/or PPARδ expression vectors, was transfected into the quadriceps muscle of C57BL6 mice. Activation of the luciferase reporter gene was measured in relative light units and normalized to dual luciferase activity. Mean values from experiments (n = 5) are shown as fold induction, where the activity in the absence of RXRγ is the reference value (set at 100). (*B*) Schematic representations of serial deletion of *Glut1* promoter constructs are shown in the figure. Squares denote the putative PPAR/RXR binding sites. Open bars; *Glut1*-Luc without RXRγ and PPARδ expression vectors, and filled bars; *Glut1*-Luc with RXRγ and PPARδ expression vectors. The activity in the absence of RXRγ and PPARδ in each experiment for different *Glut1*-Luc construct in the reference value (set at 100). ** *P*<0.01, compared with the value of wild-type promoter in the absence of RXRγ/PPARδ.

To examine whether RXRγ directly activates mRNA expression of *Glut1*, we performed the *in vivo* luciferase reporter analysis using *Glut1* promoter (*Glut1*-Luc). In this study, the activity of *Glut1*-Luc was marginally enhanced by RXRγ alone (Fig. 4*A*). Moreover, we found no significant activation of the *Glut1*-Luc by PPARδ, an important regulator of glucose as well as lipid metabolism in the skeletal muscle [7]. Interestingly, combination of RXRγ and PPARδ resulted in significant increase in *Glut1*-Luc activity in the skeletal muscle *in vivo* (*P*<0.01) (Fig. 4*A*). Motif search analysis revealed two putative PPAR-responsive elements (PPRE1 and PPRE2) (−657/−645 and −520/−508, respectively) in the mouse *Glut1* promoter. A series of deletion mutant analysis showed that combination of RXRγ and PPARδ activates the regions of −1500/+75, and −647/+75, but not the region of −152/+75 in the *Glut1* promoter (Fig. 4*B*), suggesting that the second putative PPRE (−520/−508) is involved in the RXRγ/PPARδ-induced *Glut1* promoter activation.

### Microarray and bioinformatics analyses of up-regulated gene in RXRγ mice

**Figure 5:**
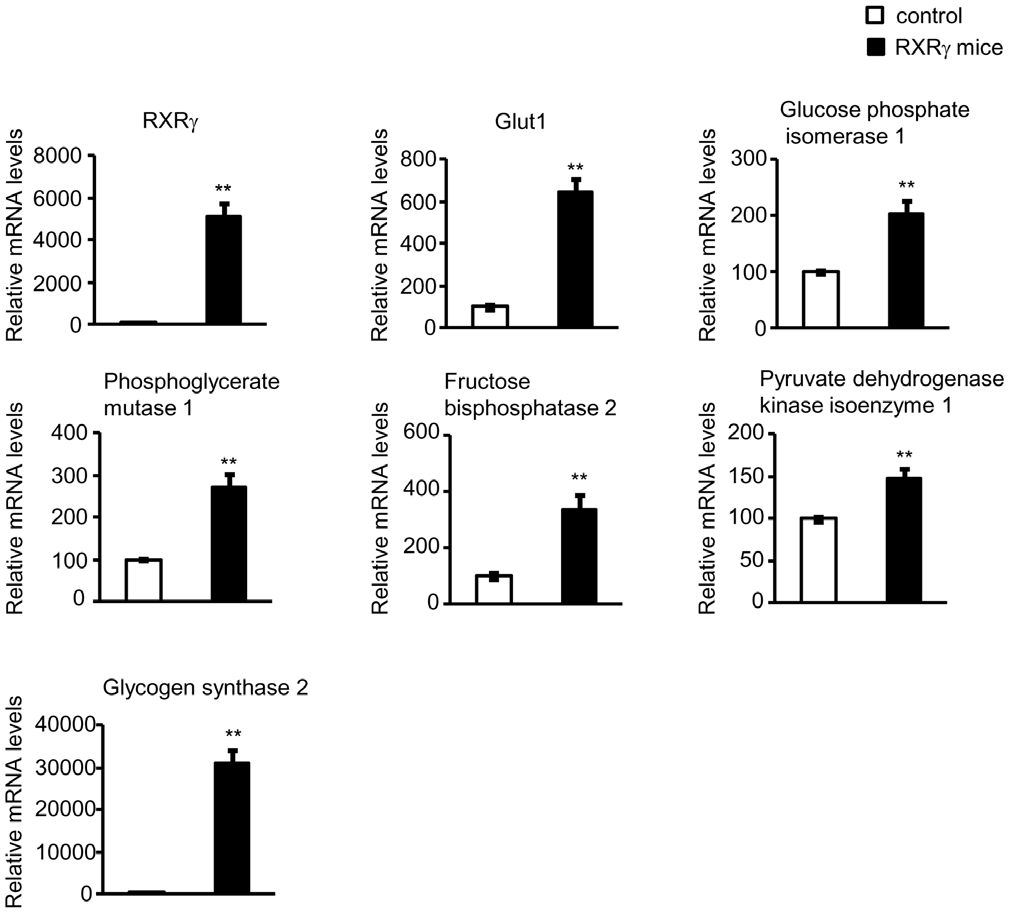
Levels of ‘glucose metabolic process’ gene expression in the skeletal muscle of RXRγ mice. Representative gene expressions of ‘glucose metabolic process genes’ analyzed by microarray and GO analysis (Table 5) were examined by quantitative real-time PCR. The value for wild-type (littermates of line 4-3) mice was set at 100, and relative values are shown. Mice were females of 4 months of age. The number of animals was 6 for both control (open bars) and RXRγ (filled bars) mice. These samples were also used in microarray analysis (Dataset S1). * *P*<0.05 and ** *P*<0.01 compared with respective control. N. S., not significant.

In order to gain insight into the gene expression change in RXRγ mice, we performed microarray analysis. As shown in Dataset S1, 738 genes were up-regulated in the analysis. As expected, Glut1 expression was increased in the microarray data. Also, SREBP1c expression, which we previously reported [9], was increased in the microarray data. Using the data, we performed GO analysis to determine if genes, up-regulated in RXRγ mice, are associated with particular biological processes. Our GO analysis revealed genes with increased expression in the RXRγ mice in various categories (Table 4), including ‘glucose metabolic process’ genes and ‘fatty acid biosynthetic process’ genes, which indicated that overexpression of RXRγ affects the expression of many genes. The up-regulated genes categorized as glucose metabolism genes in GO term are listed in Table 5. Among them, we confirmed enhanced gene expression by quantitative real time PCR (Fig. 5), supporting the microarray data reliable. Moreover, we calculated the ratio of putative transcription factor binding motifs in glucose metabolism genes, which were up-regulated in RXRγ mice. In the sample, several motifs showed statistical significance (Table 6) (*P*<0.05), including PPAR responsive elements. These data suggest that glucose metabolism genes up-regulated in RXRγ mice are possible target genes of the RXRγ and PPAR heterodimer.

**Table 4:** Gene Ontology Analysis.

GO ID	GO Term	P value
GO:0060537	muscle tissue development	6.91E-05
GO:0006461	protein complex assembly	6.78E-05
GO:0008104	protein localization	1.18E-04
GO:0051146	striated muscle cell differentiation	6.06E-04
GO:0030334	regulation of cell migration	8.04E-04
GO:0006886	intracellular protein transport	0.002367379
GO:0019220	regulation of phosphate metabolic process	0.00312153
GO:0048514	blood vessel morphogenesis	0.00172443
GO:0045859	regulation of protein kinase activity	0.00563927
GO:0006915	apoptosis	0.007513423
GO:0006006	glucose metabolic process	0.002958786
GO:0006917	induction of apoptosis	0.005257921
GO:0042981	regulation of apoptosis	0.014518039
GO:0043066	negative regulation of apoptosis	0.022995692
GO:0006633	fatty acid biosynthetic process	0.012664127
GO:0032956	regulation of actin cytoskeleton organization	0.027565775
GO:0043388	positive regulation of DNA binding	0.021966247
GO:0006469	negative regulation of protein kinase activity	0.045894089
GO:0016477	cell migration	0.045248711
GO:0030521	androgen receptor signaling pathway	0.027402985
GO:0055003	cardiac myofibril assembly	0.020081231
GO:0000165	MAPKKK cascade	0.032045331
GO:0046825	regulation of protein export from nucleus	0.027402985

738 genes up-regulated in RXRγ mice compared with wild-type mice by microarray (Listed in Dataset S1) were classified into GO functional annotations, as described in Methods.

**Table 5:** List of ‘glucose metabolic process’ genes.

Gene Symbol	Gene description
Atf3	Activating transcription factor 3
Bpgm	2-3-bisphosphoglycerate mutase
Dcxr	Dicarbonyl L-xylulose reductase
Fbp2	Fructose bisphosphatase 2
Gbe1	Glucan branching enzyme 1
Gpd1l	Glycerol-3-phosphate dehydrogenase 1-like
Gpi1	Glucose phosphate isomerase 1
Gys2	Glycogen synthase 2
Igf2	Insulin-like growth factor 2
Mat2b	Methionine adenosyltransferase II, beta
Nisch	Nischarin; an imidazoline receptor
Pdk1	Pyruvate dehydrogenase kinase isoenzyme 1
Pgam1	Phosphoglycerate mutase 1
Pgd	Phosphogluconate dehydrogenase
Pgm2l1	Phosphoglucomutase 2-like 1
Phkb	Phosphorylase kinase beta

Up-regulated genes in RXRγ mice in the microarray, classified as ‘glucose metabolic process’ genes by GO analysis, as described in Method. Genes are listed in alphabetic order of gene symbol. Glut1, which appeared in the up-regulated list in the microarray (Dataset S1), is not included in this list, as it was classified ‘transporter’ in the GO analysis.

**Table 6:** Possible transcription factor binding sites in the ‘glucose metabolic process’ gene up-regulated in RXRγ mice.

Transcription factors	Matrix name	P-value
WT1, WT1 -KTS, WT1 I, WT1 I -KTS	WT1_Q6	6.98E-04
HNF-4, HNF-4alpha	HNF4ALPHA_Q6	0.0013
PPARalpha	PPARA_01	0.0017
c-Myb, c-Myb-isoform1	VMYB_02	0.0026
PPARalpha, PPARdelta, PPARgamma	PPAR_DR1_Q2	0.003
CART1, CART1, CART1	CART1_01	0.0036
COUP-TF1, COUP-TF2, HNF-4, HNF-4alpha	COUP_DR1_Q6	0.0036
CP2, CP2-isoform1	CP2_02	0.0055
NRF1-isoform1, NRF1-isoform2, NRF1-xbb1	TCF11_01	0.0057
SZF1	SZF11_01	0.0057
PPARgamma	PPARG_01	0.0057
COUP-TF1, COUP-TF2, HNF-4, HNF-4alpha, HNF4gamma	DR1_Q3	0.0057
BRCA1, BRCA1	BRCA_01	0.0065
HNF-3beta	HNF3B_01	0.007
Pax-2b, pax2	PAX2_01	0.007
C/EBPalpha, C/EBPbeta(LAP), C/EBPbeta(p20), C/EBPbeta(p20), C/EBPbeta(p34), C/EBPbeta(p35), C/EBPgamma, cebpe, CRP3, NF-IL6-1, NF-IL6-3	CEBP_Q3	0.007
MITF, MITF-M1, tcfec, TFEA, TFEA-xbb1, TFEA-xbb2, tfeb, tfeb-isoform1	TFE_Q6	0.007
AP-2alpha, AP-2beta, AP-2gamma	AP2_Q6_01	0.0077
Pax-4a, Pax-4c, Pax-4d, Pax4	PAX4_04	0.0079
c-Myc, deltaMax, max, max-isoform1, max-isoform2, N-Myc	MYCMAX_03	0.0093

The mouse genome in the region of −1000 to +100 relative to the transcription start of an individual gene classified as glucose metabolism gene by GO analysis (Table 5), was analyzed. Statistical hypothesis testing was evaluated against housekeeping genes of mice. Matrics names are based on the MATCH software (see **Methods**), which are listed in *P*-value order. Indicated binding sites of transcription factors appeared significantly more frequent occurrence in the promoter sets.

## Discussion

RXRγ is a nuclear receptor-type transcription factor that is expressed abundantly in the skeletal muscle and is regulated by nutritional conditions. Treatment of obese and diabetic mice with RXR pan-agonists (agonists for all the RXR isoforms) has improved glucose metabolism in mice [21]–[23], suggesting the beneficial effect of RXR on diabetes. However, which RXR isoform(s) are involved and where they work to improve diabetes has not been addressed. Here, we investigated glucose metabolism in RXRγ mice.

We demonstrated increased glucose metabolism in RXRγ mice relative to control mice, although there was no significant difference in the insulin-induced hypoglycemic effect between genotypes, based on insulin tolerance test and glucose clamp analysis. Because insulin level is similar between RXRγ mice and control mice, it is unlikely that increased insulin secretion is responsible for increased glucose tolerance in RXRγ mice. On the other hand, mRNA and protein expression of Glut1 is increased in the skeletal muscle from RXRγ mice relative to control mice, with increased glucose uptake and glycogen content. Because transgenic overexpression of Glut1 in the skeletal muscle has resulted in increased glucose uptake [24], glycogen content [25] and lowering of blood glucose [24], it is likely that enhanced glucose tolerance in RXRγ mice is mediated at least in part by increased Glut1 in the skeletal muscle.

As RXRγ is a nuclear receptor-type transcription factor that heterodimerizes with many nuclear receptors [6], we examined whether the *Glut1* gene is directly regulated by RXRγ. In an *in vivo* luciferase reporter analysis, combination of RXRγ and PPARδ activates the *Glut1* promoter activity, which is diminished by deletion of the putative PPREs. It is, therefore, RXRγ/PPARδ may activate *Glut1* expression in the skeletal muscle *in vivo*. In this regard, we found that overexpression of RXRγ or PPARδ alone or both in C2C12 myocytes *in vitro* does not induce *Glut1* gene expression (unpublished data). This may be because C2C12 cells lack other factor(s) that are present in the skeletal muscle *in vitro* and are required to activate *Glut1* transcription. Whether the increased *Glut1* gene in RXRγ mice is mediated by RXRγ/PPARδ should be confirmed by additional experiments using PPARδ knockout mice. Meanwhile, the transgene expression level in RXRγ mice was very high, and appeared to be beyond the physiological level. Nonetheless, if RXRγ can be enhanced in skeletal muscle, an increase in glucose metabolism can be expected.

We recently demonstrated that in addition to glycogen content, RXRγ mice exhibit increased triglyceride in the skeletal muscle as a result of increased SREBP1c gene expression [9]. In obese and diabetic subjects, intramuscular lipid is high with insulin resistance [26]. Moreover, in athletes, the skeletal muscle is also high in lipid but with high insulin sensitivity, which is known as the athlete paradox [27], [28]. A partial explanation is an increased fatty acid load in obese and diabetic subjects, but not in athletes; increased intramuscular lipotoxic fatty acid metabolites, such as diacylglycerol and ceramide, cause insulin resistance in skeletal muscles [29], [30]. Taken together with our previous report [9], this study demonstrates that RXRγ mice do not develop insulin resistance despite high intramuscular lipids, and appear to be protected against obesity-induced lipotoxicity in the skeletal muscle. Depositing glucose as both glycogen and triglycerides in the skeletal muscle may be effective in enhancing glucose metabolism.

Microarray analysis showed that various genes are up-regulated in the skeletal muscle of RXRγ mice. As RXRγ can heterodimerize several nuclear receptors, it is not surprising that expression of many genes was up-regulated by RXRγ overexpression. Concerning glucose metabolism genes, in the skeletal muscle of RXRγ mice, the expression levels of genes, such as glucose phosphate isomerase 1 and phosphoglycerate mutase 1 (stimulate glycolysis or gluconeogenesis), fructose bisphosphatase 2 (stimulates gluconeogenesis), pyruvate dehydrogenase kinase isoenzyme 1 (suppresses glycolysis), glycogen synthase 2 (stimulates glycogen synthesis)[31], [32], were markedly increased (Fig. 5), suggesting the anabolic reaction of glucose. This is consistent with the observation that the glycogen content of skeletal muscle is higher in RXRγ mice (Fig. 2C). It is of note that glycogen synthase 2 is a liver-type enzyme, not a skeletal muscle-type enzyme [32]. In addition, usually, gluconeogenesis is not considered to be a major metabolic pathway in skeletal muscle. Chronic transgenic overexpression of RXRγ may have caused a super-physiological gene expression change in the skeletal muscle. Meanwhile, our bioinformatics analysis showed that glucose metabolism genes up-regulated in RXRγ mice contain several transcription factor binding motifs including PPRE in their promoter region. These observations support the concept that the RXRγ/PPAR heterodimer contributes to activation of these gene sets, although this needs to be confirmed by further experiments.

In summary, we demonstrated enhanced glucose metabolism with increased Glut1 expression and glucose uptake in RXRγ mice. This study suggested that activation of the skeletal muscle RXRγ is a novel therapeutic strategy to treat or prevent type 2 diabetes.

## Supporting Information

Dataset S1 **List of genes up-regulated in RXRγ mice compared with wild-type control mice by microarray.**
(XLS)
